# Supplementary material for: Export trade structure transformation and countermeasures in the context of reverse globalization
Source: PLoS One. 2022 Jun 24;17(6):e0270390. doi: 10.1371/journal.pone.0270390 (PMC9231787; doi:10.1371/journal.pone.0270390)
Supplement: S1 Appendix — (PDF) [file pone.0270390.s001.pdf]

S1 Appendix. List of 42 countries and one region.

Serial number	Country or region	ISO 3	Location	Serial number	Country or region	ISO 3	Location
1	Australia	AUS	Oceania	23	Ireland	IRL	Europe
2	Austria	AUT	Europe	24	Italy	ITA	Europe
3	Belgium	BEL	Europe	25	Japan	JPN	Asia
4	Bulgaria	BGR	Europe	26	South Korea	KOR	Asia
5	Brazil	BRA	South America	27	Lithuania	LTU	Europe
6	Canada	CAN	North America	28	Luxembourg	LUX	Europe
7	Switzerland	CHE	Europe	29	Latvia	LVA	Europe
8	China	CHN	Asia	30	Mexico	MEX	North America
9	Cyprus	CYP	Asia	31	Malta	MLT	Europe
10	Czech Republic	CZE	Europe	32	Netherlands	NLD	Europe
11	Germany	DEU	Europe	33	Norway	NOR	Europe
12	Denmark	DNK	Europe	34	Poland	POL	Europe
13	Spain	ESP	Europe	35	Portugal	PRT	Europe
14	Estonia	EST	Europe	36	Romania	ROU	Europe
15	Finland	FIN	Europe	37	Russian Federation	RUS	Europe
16	France	FRA	Europe	38	Slovakia	SVK	Europe
17	Great Britain	GBR	Europe	39	Slovenia	SVN	Europe
18	Greece	GRC	Europe	40	Sweden	SWE	Europe
19	Croatia	HRV	Europe	41	Turkey	TUR	Asia
20	Hungary	HUN	Europe	42	Taiwan	TWN	Asia
21	Indonesia	IDN	Asia	43	United States of America	USA	North America
22	India	IND	Asia				
