# Supplementary material for: Export trade structure transformation and countermeasures in the context of reverse globalization
Source: PLoS One. 2022 Jun 24;17(6):e0270390. doi: 10.1371/journal.pone.0270390 (PMC9231787; doi:10.1371/journal.pone.0270390)
Supplement: S1 Table — (PDF) [file pone.0270390.s002.pdf]

S1 Table. Goodness of fit test results under different variable forms.

Statistics	C	GDP	EXP	C	$\log(GDP)$	$\log(EXP)$
t	97.1128	-4.5245	0.3593	20.5583	-7.9426	2.0212
Prob.	0.0000	0.0003	0.7240	0.0000	0.0000	0.0603
R-squared		0.9633			0.9875	
Ad R-squared		0.9587			0.9860	
